# Optimal use of the FDG-PET/CT in the diagnostic process of fever of unknown origin (FUO): a comprehensive review

**DOI:** 10.1007/s11604-022-01306-w

**Published:** 2022-07-04

**Authors:** Ryogo Minamimoto

**Affiliations:** Division of Nuclear Medicine, Department of Radiology, National Center for Global Health and Medicine, 1-21-1, Toyama, Shinjyuku-ku, Tokyo, 162-8655 Japan

**Keywords:** Fever of unknown origin, FUO, FDG-PET/CT, IUO

## Abstract

Numerous studies have clarified the usefulness of ^18^F-fluorodeoxyglucose (FDG)-PET/CT (positron emission tomography) for diagnosing the cause of fever of unknown origin (FUO). Various types of disease can cause FUO, but the cause remains unknown in a certain proportion of FUO, even when the advanced diagnostic methodologies are used. FDG-PET/CT is regarded as a second-line modality in the diagnostic process of FUO, and its potential to identify the cause of FUO will be maximized when the appropriate clinical considerations are understood. Accordingly, this review presents basic knowledge regarding FUO, and reports the current status of FDG-PET/CT applied to diagnosing the cause of FUO, including diagnostic performance, test protocols, possible factors influencing the diagnostic result, outcomes, and cost-effectiveness. This knowledge will enable effective future use of FDG-PET/CT to improve outcomes in patients with FUO.

## Introduction

In 1961, Petersdorf and Beeson defined fever of unknown origin (FUO) as "a condition in which fever of 38.3 °C or higher as sublingual temperature is observed several times for more than 3 weeks, and the cause is unknown even after hospitalization for 1 week or longer" [1]. In 1991, Durack and Street revised the criteria as "a condition in which fever of 38.3 °C from unknown origin persists for more than 3 weeks and cannot be diagnosed by 3 days of inpatient examination or 3 outpatient examinations” [2]. FUO based on these two criteria is termed “classic FUO”. FUO is a common manifestation of multiple disparate disease processes that can be subcategorized into classic FUO, nosocomial FUO (FUO that develops in hospitalized persons), immunodeficiency-associated FUO, and travel-associated FUO. More recently, FUO has been defined as (1) fever ≥ 38.3 °C (≥ 101 ℉) on at least two occasions, (2) illness duration of ≥ 3 weeks, (3) no known immunocompromised state, (4) uncertain diagnosis even after thorough history-taking, physical examination, and the following obligatory investigations: determination of erythrocyte sedimentation rate (ESR) and C-reactive protein (CRP) level, platelet count, leukocyte count and differential, measurement of levels of hemoglobin (Hb), electrolytes, creatinine, total protein, alkaline phosphatase, alanine aminotransferase, aspartate aminotransferase, lactate dehydrogenase, creatine kinase, ferritin, antinuclear antibodies, and rheumatoid factor; protein electrophoresis, urinalysis, blood cultures, urine culture, and chest X-ray; abdominal ultrasonography (AUS), and tuberculin skin test or interferon γ release assay [3]. As clinical studies of FDG-PET or FDG-PET/CT for the evaluation of FUO have mostly employed the definition of classic FUO using either the Petersdorf and Beeson or Durack and Street criteria, this review therefore focuses on classic FUO.

The majority of diseases that cause FUO are well known and are discussed later in this review. Although evidence-based diagnostic criteria and guidelines have been established for these diseases, FUO describes the state in which the background disease cannot be identified by the diagnostic workup. In general, unknown cause of prolonged fever leads to limitations in daily life that include various physical symptoms, mental load, and total debilitation that may be related to social and economic losses. Considering the known severity and prognosis of the various diseases that cause FUO, irreversible disorders or death can occur if left untreated.

When the cause of FUO is not identified through the diagnostic workup, FDG-PET/CT can be a gamechanger for guiding a final diagnosis. However, it should be used efficiently in the clinical situation due to its limitations of radiation exposure and the high cost.

## Mechanism of fever

Fever is an active increase in body temperature and is regulated by the hypothalamus. When a bacterium or virus invades the body, it is recognized by Toll-like receptors (TLRs) present in macrophage cells and dendritic cells. When TLRs are stimulated, inflammatory cytokines such as interleukin (IL)-1β and IL-6 are released, which stimulate the production of acute phase proteins such as CRP, fibrinogen, and serum amyloid A protein. When inflammatory cytokines produced in peripheral tissues act on cytokine receptors in cerebrovascular endothelial cells, they promote the production of prostaglandin E2 (PGE2) which is a mediator of fever. When PGE2 acts on the EP3 receptor in the preoptic area (POA), which is the center of thermoregulation, the thermogenic reaction is enhanced and the heat dissipation reaction is suppressed [4–8].

In addition to cerebrovascular endothelial cells, fever is also caused by PGE2 produced by macrophages in the lungs and liver, afferent signals from vagus nerves and somatosensory nerves located locally in inflammation, and activation of microglia and astrocytes caused by peripheral inflammation. Non-shivering heat production and cutaneous vasoconstriction in brown adipose tissue, which play major roles in the exothermic reaction, are caused by increased sympathetic nerve activity. The sympathetic nerves involved in fever are the dorsomedial hypothalamus (DMH), the rostral ventromedial part of the medulla oblongata centered on the rostral nucleus raphe plaque and the large raphe nucleus, and the thoracic spinal cord to the upper lumbar spinal cord, due to excitement of neurons at the level of the intermediolateral nucleus (IML) of the spinal cord [5]. Neurons with persistent inhibitory control project from the POA to the DMH and the ventromedial part of the medulla oblongata, but when PGE2 acts on the EP3 receptor of POA, the activity of POA neurons decreases. Thus, fever is caused by release of the brake of the POA on the DMH and the sympathetic nervous system by PGE2 [4]. Fever can be caused not only by infectious diseases but also by autoimmune diseases, malignant tumors, tissue disorders, such as surgical invasion and myocardial infarction, and cytokine therapy related to PGE2 activity [9]. Abundant inflammatory cells such as neutrophils and macrophages can exist in an area that is the cause of fever, and can thus be identified by FDG-PET/CT as accumulation in these inflammatory cells.

## Possible responsible causative diseases of FUO and their trends

FUO can be caused by a wide group of diseases, including both benign and serious conditions. The main categories of diseases responsible for FUO are infections, noninfectious inflammatory disease (NIID), neoplasm (mainly malignancy), and miscellaneous others. Among bacterial infections, tuberculosis is the most common infectious cause of FUO [10], and others include infective endocarditis, abscess, prostatitis, Whipple’s disease, and typhoidal and nontyphoidal salmonella serovars [3]. Viral infections that can cause FUO include cytomegalovirus, Epstein–Barr virus (EBV), human herpesvirus (HHV)-6, and HHV-7 [11]. EBV viremia is characterized by fever with hematologic abnormalities. The clinical presentation of infectious mononucleosis can vary with age. HHV-6 and HHV-8 should generally be tested only in immunocompromised patients [12]. Fungal infections, such as aspergillosis and cryptococcosis, tend to occur in immunocompromised persons [10]. Neoplasms commonly associated with FUO, based on pyrogenic cytokine production or spontaneous tumor necrosis, include lymphomas, renal-cell carcinoma, hepatocellular and ovarian cancer, atrial myxoma, and Castleman’s disease [8, 13]. Causative NIIDs consist mainly of autoinflammatory and autoimmune disorders, alone or in combination. Autoinflammatory conditions are disorders of innate immunity with dysregulated interleukin-1β responses, interleukin-18 responses, or both. Autoimmune diseases involve adaptive immunity and are driven by a type 1 interferon response [14]. Adult-onset Still’s disease (AOSD) and rheumatoid arthritis (RA), giant-cell arteritis (GCA), and polymyalgia rheumatica (PMR) are associated diseases. The major possible cause of FUO that is categorized as ‘miscellaneous’ is drug-associated fever [15].

According to a summary of clinical studies on FUO, the classification of diseases causing FUO differs between western countries and others. In western counties, the ratios of classifications causing FUO were 19, 24, 12, 8, and 38% for infections, NIID, malignancy, miscellaneous, and unknown, respectively; whereas in other countries, these have been reported as 43, 20, 14, 7, and 16%, respectively. The spectrum of diseases causing FUO has changed due to the widespread use of antibiotics and to new and improved diagnostic techniques such as CT, AUS, and echocardiography that contribute to early detection of the source. The major difference in causes of FUO between western counties and others is the proportion of infectious diseases, primarily tuberculosis. In western countries, the underlying cause of FUO in undiagnosed cases remains difficult to determine, because the use of advanced diagnostic techniques has enabled early diagnosis of FUO and thus earlier consultation in the majority of cases [3].

In children, infections are the major cause of FUO, accounting for 51% of cases according to a meta-analysis based on 18 studies [16, 17]. The trend in infections differs between developed countries, where bacterial infections, EBV, and Bartonella are representative; and that in developing countries, in which brucellosis, typhoid fever, tuberculosis, rickettsia infections, and abscesses are representative. Although malignancy occurs less frequently in children than in adults, lymphoma and leukemia are major causes of FUO in children, as is the case in adults. Autoimmune/rheumatologic conditions are also less frequent in children than in adults, and IBD, Crohn’s disease, and the systemic form of juvenile idiopathic arthritis are particular diseases that may cause FUO in children. Approximately 25–30% of children with FUO remain undiagnosed through the diagnostic workup for FUO, which is the same trend as in adults [16–18].

**Fig. 1:**
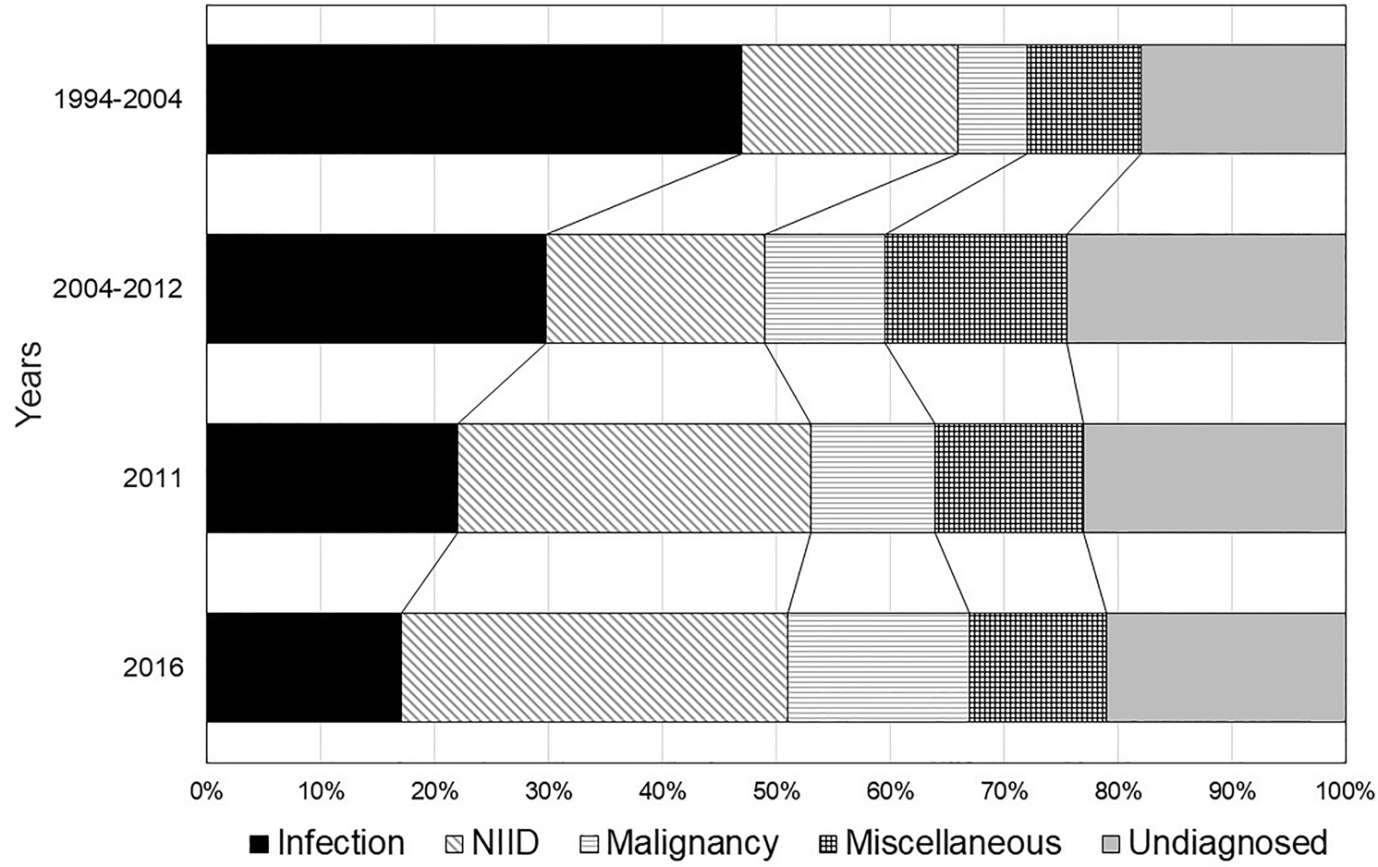
Transition of disease types causing FUO in adult patients in Japan. Infectious disease is the major cause of FUO, but has decreased with time. Noninfectious inflammatory disease (NIID) has increased gradually as the cause of FUO. The ratio of FUO caused by malignancy has not changed in recent 10 years. Approximately 20% of FUO has ended with undiagnosed

Figure 1 shows the transition of disease types causing FUO in adult patients in Japan [19–22]. Infectious disease was the major cause of FUO prior to 2004, but has decreased with time, along with gradual increases in NIID. The ratio of FUO caused by malignancy has not changed in recent 10 years. Undiagnosed cases have shown variation, but it has occurred at a large scale and constant rate. According to the latest survey in Japan (FDG-PET utilization rate, 31.2%), the major causes of FUO are lymphoma (8%), AOSD (5%), PMR (4%), vasculitis (4%), viral infection (4%), pericarditis (3%), and RA (3%). The number of cases of malignancy has increased in patients aged ≥ 65 years, half of which were malignant lymphoma [22].

## Prognosis of patients with FUO

As FUO is caused by a variety of diseases, the overall prognosis depends on the background disease [23]. The mortality of immunocompetent patients with FUO in Belgium was 21–33% prior to 1980, decreasing to 6.5–16% during 1980–2000 and then to approximately 7% in the 2000s [24]. In Japan, the corresponding mortality rate was reported as 7% (9/121) in 2016–2017 [22]. Among the various causes of FUO, malignancy has the highest association with mortality. In the 2000s, the malignancy-associated mortality rate was 38–40%, which is much lower than that prior to 1980 (52–89%). Improvements in screening methods and the performance of diagnostic tools for malignancy might have been instrumental in the gradual decrease in mortality over time [24]. Lymphoma presenting as FUO tends to have a rapid progression and poor prognosis, and is difficult to diagnose [25]. Differences in prognosis among other diseases that cause FUO, with and without prolonged fever, are unknown. The prognosis of patients with FUO caused by NIID is a remaining issue. Considering the reduction in mortality during a half-century, improvements in screening methods and the establishment of clinical guidelines have enabled diagnosis of diseases with high mortality ratios. Cases without a final diagnosis may be caused by unknown diseases or a focus of fever that is undetectable even using current medical technology.

Tan studied the prognosis of 58 patients in 2004–2010, and reported that in 35 of 47 cases (74.5%) of undiagnosed FUO, the fever subsided during hospitalization or after discharge. Two patients required continued treatment with anti-inflammatory drugs and 10 patients (21.3%) died during follow-up, with 9 deaths caused by severe and worsening conditions related to the febrile illness [26]. Mulders-Manders et al. reported the prognosis of 131 cases of undiagnosed FUO among 274 patients with FUO, and found remission of fever in 47.3%. In this cohort, 66.7% of patients showed improvement by empirical treatment. Mortality occurred in 6.9% of the cohort, but appeared to have no relation with the febrile disease in most cases [27].

In FUO patients for whom a final diagnosis could not be obtained regardless of extensive investigation, prognosis was generally good and mortality was low [26, 27]. The same trend was confirmed in another study that found that patients with unexplained fever did not have serious illness [28]. In addition, 43–75% of adult patients with undiagnosed FUO experience spontaneous remission of fever [29–32], and empirical treatment with NSAIDs or corticosteroids increases this proportion [23].

Empirical administration of antimicrobial or anti-inflammatory therapy has been attempted blindly in patients with prolonged fever. For patients with unexplained FUO, antibiotics, NSAIDs, corticosteroids, and anakinra are major empirical treatments, and have been reported as effective in 60–66.7% of patients [26, 27]. However, therapeutic antimicrobial trials may cause a predisposition to resistance or suppress the growth of fastidious pathogens, and such treatment can be misleading as to the underlying cause of fever. Therefore, every possible attempt should be made to establish the diagnosis unless there is a rapid deterioration in clinical status [33].

## Principles of FDG-PET/CT

FDG is a glucose-like substance in which the hydroxyl group at the 2-position of glucose is replaced with ^18^F, which is a positron-emitting nuclide. Like glucose, FDG is taken up into cells via glucose transporters and is phosphorylated by hexokinase, but, unlike glucose, is not metabolized by glycolysis and accumulates in the cell [34, 35]. In inflammatory cells, the glucose transporters GLUT1 and GLUT3 in the cell membrane are increased in the same manner as in malignant cells, and it is thought that glucose utilization is enhanced accordingly. In inflammation and infected foci, glucose consumption by activated inflammatory cells is 10 times greater than that in the inactivated state, which is the mechanism by which inflammatory tissue is visualized as high FDG accumulation [36–38].

Based on this biological background, FDG-PET and PET/CT are approved for use in Japan and are covered by national health insurance for staging of malignancy (except for early stage gastric cancer), screening for recurrence and metastasis of malignancy, and for diagnosis of the active lesion in cardiac sarcoidosis and large vessel aortitis (Takayasu arteritis and GCA).

As FDG shows high accumulation in both inflammatory diseases and malignant tumors, FDG-PET/CT examination enables identification of a causative focus of prolonged fever that is undetectable by other diagnostic imaging modalities. Even in situations where it is difficult to accurately identify a specific disease, FDG-positive areas have high probability of containing cancer and inflammatory cells, and thus, the pathological diagnosis is confirmed by accurate biopsy or puncture and the pathological diagnosis can be confirmed at an early stage, leading to early treatment of the FUO. Based on this mechanism, numerous clinical studies have demonstrated the utility of FDG-PET or FDG-PET/CT for the diagnosis of FUO.

## Diagnostic workup for patients with FUO

The crucial process in the diagnostic workup in patients with FUO is to search for potential diagnostic clues (PDC), such as localizing signs, symptoms, and abnormalities pointing toward a diagnosis, through complete and repeated history-taking, and physical examination and obligatory investigations [3]. In addition, considering discontinuation or replacing medication to exclude drug fever is an important process.

**Fig. 2:**
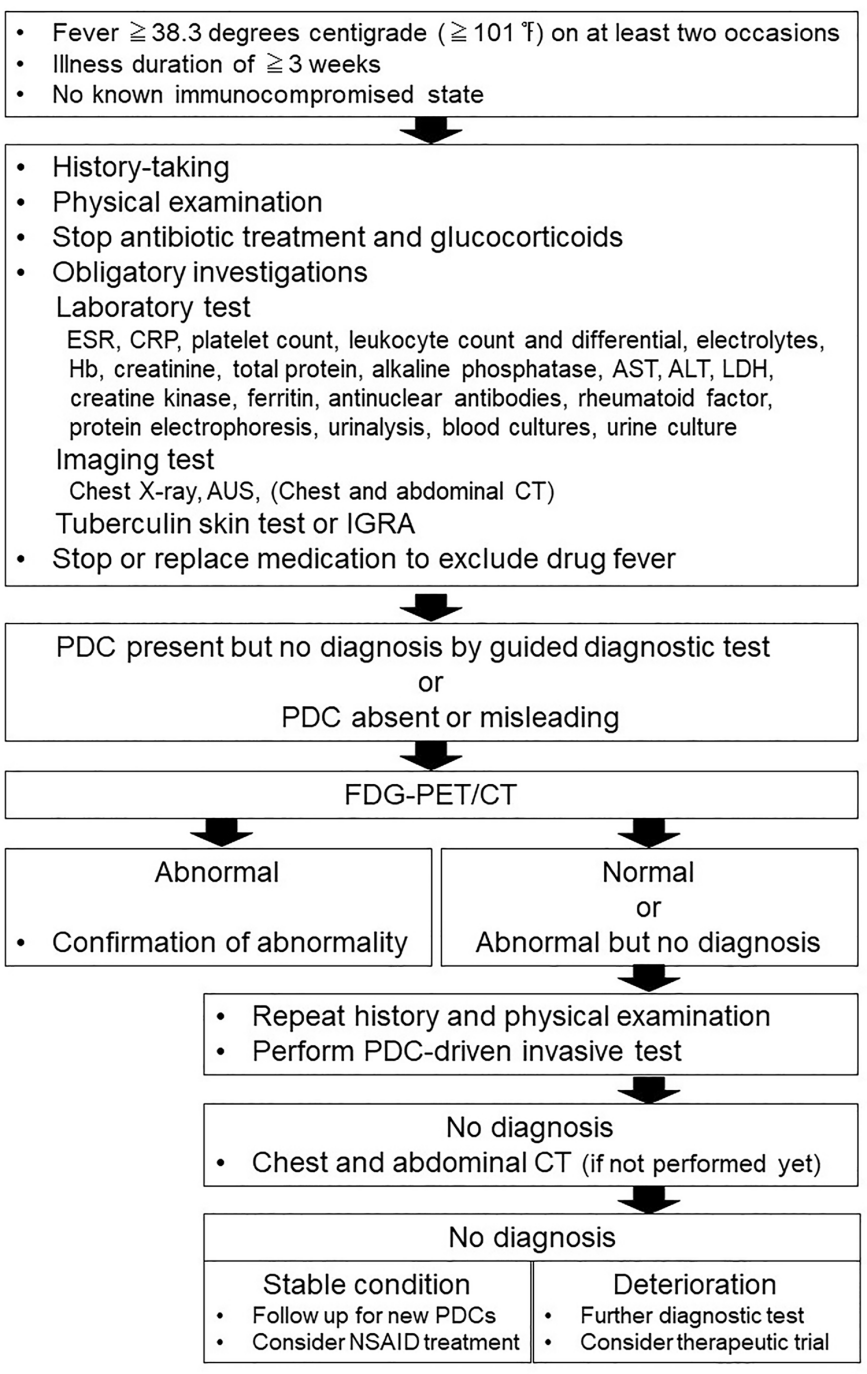
Diagnostic flow for FUO modified from Harrison’s principles of internal medicine. *ESR* erythrocyte sedimentation rate, *CRP* C-reactive protein, *Hb* hemoglobin, *AST* alanine aminotransferase, *ALT* aspartate aminotransferase, *LDH* lactate dehydrogenase, *AUS* abdominal ultrasonography, *IGRA* r interferon γ release assay, *PDC* potential diagnostic clues, *NSAID* Non-Steroidal Anti-Inflammatory Drugs

Figure 2 shows the diagnostic flow for FUO, which is modified from what is presented in Harrison’s principles of internal medicine [3]. Laboratory tests and imaging examinations are mainstream in the diagnostic process of FUO. Despite the high false-positive rate of US and low sensitivity of chest X-ray, these low-cost examinations remain obligatory in the diagnostic workup to separate patients in whom the cause of FUO is easily diagnosed. These are standard and widely performed examinations that can generally be completed in an outpatient visit. In the suggested flow of the diagnostic process of FUO, CT examination is regarded as later stage diagnostic test, along with bone marrow, temporal artery, and liver biopsies. The diagnostic yield of screening chest and abdominal CT in patients with FUO is low (< 20%), but the specificity is high. Although CT has limited additional value after a normal FDG-PET/CT, CT is a minimally invasive examination that may contribute to the diagnosis due to its high sensitivity [3]. However, according to clinical studies shown in later section, chest and abdominal CT examinations are frequently performed prior to scintigraphy or FDG-PET/CT. Considering the penetration rate and the cost, CT examinations have more accessibility than scintigraphy and FDG-PET/CT in the current clinical situation.

## Performance of scintigraphy for the diagnosis of FUO

The basic concept of nuclear imaging is to noninvasively detect areas of functional changes in the total body. Representative nuclear imaging for the diagnosis of FUO has been ^67^Ga-citrate scintigraphy and ^111^In or ^99m^Tc labeled leukocyte scintigraphy (LS) [3]. The sensitivity, specificity, and diagnostic yield of ^67^Ga-citrate scintigraphy was summarized based on 6 studies with 397 cases as 60% (95% CI 0.45–0.73), 63% (95% CI 0.37–0.84) and 35% (95% CI 0.25–0.46), respectively [39]. A direct comparison between FDG-PET/CT (sensitivity: 79%, specificity: 56%, accuracy: 72%, positive predictive value [PPV]: 83%, and negative predictive value [NPV]: 36%) and Ga single photon emission computed tomography (SPECT) /CT (sensitivity: 45%, specificity: 81%, accuracy: 55%, PPV: 86%, and NPV: 50%) revealed that FDG-PET/CT showed higher sensitivity and accuracy, and lower specificity, than Ga SPECT/CT. The positive contribution of FDG-PET/CT (72%) was higher than that of Ga SPECT/CT (55%). A high false-positive rate of 44% was observed for FDG-PET/CT, whereas a high false-negative rate of 55% was observed for Ga SPECT/CT [40]. Another prospective multicenter study showed the same trend (FDG-PET/CT: sensitivity: 45%, specificity: 40%, accuracy: 44%, PPV: 67%, and contribution: 33%; Ga SPECT/CT: sensitivity: 25%, specificity: 72%, accuracy: 38%, PPV: 71%, and contribution: 19%) [41]. Ga SPECT requires imaging at 48 to 72 h after ^67^Ga-citrate injection, which is longer than the time required for FDG-PET/CT (delay of 1–1.5 h after intravenous FDG injection followed by approximately 30 min of scanning). The estimated radiation exposure is higher for Ga SPECT (effective dose: 0.019 mSv/MBq) than for FDG-PET (effective dose: 0.1 mSv/MBq) [42].

The sensitivity, specificity, and diagnostic yield of LS for evaluating FUO was summarized based on 6 studies with 153 cases as 33% (95% CI 0.24–0.44), 83% (95% CI 0.61–0.94), and 20% (95% CI 0.14–0.28), respectively [39]. According to a direct comparison between FDG-PET and LS, FDG-PET has a higher sensitivity than LS in identifying the etiology of FUO (FDG-PET: sensitivity: 86%, specificity: 78%, PPV: 86%, and NPV: 78%, LS: sensitivity: 20%, specificity: 100%, PPV: 100%, and NPV: 40%) [43]. In another study, FDG-PET/CT showed higher sensitivity and substantial specificity with LS (FDG-PET/CT: sensitivity: 89.7%, specificity: 73.3%; and LS: sensitivity: 66.7%, specificity: 76.9%). The odds of positive findings increased 4.6-fold for PET/CT versus LS for helping the diagnosis of FUO [44]. The limitations of LS were (1) in vitro leukocyte labeling process requires skilled personnel and is not always available, (2) the risk of radiolabeling of leukocytes with the direct manipulation of blood products, (3) patients must visit the department on two consecutive days, and the count statistic detected with LS is weak in the images at 24 h after injection, and (4) complementary bone marrow imaging is usually required to improve accuracy [45–47]. In addition, the cost of a single dose of labeled leukocytes could be as much as 8–10 times that of a single dose of FDG [44]. Radiation exposure is estimated to be approximately 14 mSv in FDG-PET/CT, which is almost the same as for labeled leukocyte SPECT/CT, approximately 13 mSv (labeled leukocyte scan alone with approximately 7–8 mSv) [47–49]. Overall, FDG-PET/CT can assume the position occupied by Ga SPECT and LS in the diagnostic process of FUO.

## Performance of FDG-PET/CT for the diagnosis of FUO

Numerous clinical studies and meta-analyses have presented the potential of FDG-PET/CT for the diagnosis of FUO. To understand the performance of FDG-PET/CT for the diagnosis of FUO, sensitivity and diagnostic yield (or contribution, as the proportion of patients in whom the imaging results were reported to contribute to the diagnosis of FUO causes) are definitely valid indexes among the various indexes applied in clinical studies. PPV is used to assess the incidence of false-positive (FP) findings in FDG-PET/CT, which can be caused by nonspecific or physiological FDG uptake. True negative (TN) cases are used to calculate specificity, NPV, and accuracy. Generally, TN is an outcome where the model correctly predicts the negative class. It is assumed that FDG-PET/CT is applied to cases of unknown cause of prolonged fever despite basic diagnostic workup. In FUO, not only does a specific reference standard not exist, but a final diagnosis might not be possible without the findings of FDG-PET/CT. Therefore, TN in FUO means a case with no abnormal findings on FDG-PET/CT in a case in which a final diagnosis was not attained through the diagnostic process, regardless that the cause of the prolonged fever was unsolved. Overall, TN is not thought of as a positive meaning, but as is presented in the previous section, true-negative cases may indicate a potentially good prognosis in patients with FUO. Therefore, the actual impacts of each of specificity, NPV, and accuracy should be reconsidered in the context of the diagnosis of FUO by FDG-PET/CT.

Ghady et al. reported that sensitivity and specificity of FDG-PET/CT for diagnosis of FUO based on four study results were 86–98% and 52–85%, respectively [33]. Kan et al. conducted a meta-analysis (based on 23 papers, 1927 cases) of FDG-PET/CT for diagnosis of FUO and reported sensitivity of 84% (95% CI 0.79–0.89), specificity of 63% (95% CI 0.49–0.75), a positive likelihood ratio of 2.3 (95% CI 1.5–3.4), and a negative likelihood of 0.25 (95% CI 0.16–0.38) [50]. Takeuchi et al. presented a meta-analysis (based on 42 papers, 2058 cases) of FDG-PET/CT for diagnosis of FUO, and reported sensitivity of 86% (95% CI 0.81–0.90), specificity of 52% (95% CI 0.36–0.37), and diagnostic yield of 58% (95% CI 0.51–0.64) [39]. Hao presented a meta-analysis (based on 15 papers, 595 cases) of FDG-PET/CT for diagnosis of FUO and reported sensitivity of 85% (95% CI 0.81–0.88) [51]. Dong et al. presented a meta-analysis (based on 9 papers, 388 cases) of FDG-PET or FDG-PET/CT for diagnosis of FUO, in which FDG-PET/CT showed sensitivity of 98.2% (95% CI 0.936–0.998), specificity of 85.9% (95% CI 0.750–0.934), positive likelihood ratio (LR) of 5.782 (95% CI 3.335–10.027), negative LR of 0.052 (95% CI 0.011–0.254), and diagnostic odds ratio (positive LR/negative LR) of 7.070 (95% CI 0.742–67.369) [52]. Bahrucha et al. found that the diagnostic yield of FDG-PET/CT was 56% (95% CI 0.50–0.61) (based on 18 papers, 905 cases) [53]. The results of these meta-analyses are in agreement that FDG-PET/CT has high sensitivity and moderate specificity for the diagnosis of FUO. Over half of patients with FUO are guided to a final diagnosis by FDG-PET/CT.

Abnormal findings on FDG-PET imaging increase the final diagnosis rate to 83%, which is higher than that of no particular findings on PET imaging, which has a rate of 36% (OR = 8.94 [95% CI 4.18–19.12]). Diagnostic performance for FUO was not influenced by PET or PET/CT, region, or follow-up period, but there was a difference between prospective (OR = 2.92 [95% CI 1.00–8.53]) and retrospective studies (OR = 18.57 [95% CI 7.57–45.59]) [54]. The higher proportion of neoplasms and infections than other causes of FUO led to a higher diagnostic yield [39].

According to another meta-analysis, FDG-PET/CT could contribute to the diagnosis of various diseases that cause FUO. The diagnostic yield of FDG-PET/CT for infectious diseases was 77.2%, among which tuberculosis (15.4%), pneumonia (9.5%), bone and joint infection (5.4%), and intra-abdominal abscess (5.0%) were the major components. The diagnostic yield of FDG-PET/CT for NIID was 64.9%, among which vasculitis (22.8%), sarcoidosis (7.0%), AOSD (5.8%), and thyroiditis (4.7%) were the major components. The diagnostic yield of FDG-PET/CT for malignancy was 95.5%, and lymphoma (61.6%) was a major component [53].

In addition to evaluation of unknown sites of inflammation, clinical indications for FDG-PET/CT in FUO patients include (1) for evaluation of unknown sites of neoplastic disease as causes of systemic symptoms, (2) to guide biopsy, (3) for assessment of therapeutic efficacy, and (4) for assessment of prognostic value [55, 56]. However, more robust evidence will be required to recommend their utility.

FDG-PET/CT has an indirect impact on the diagnosis of FUO by excluding possible causes of FUO and narrowing down the range of diseases. In a study evaluating the clinical impact of FDG-PET/CT on the clinical practice for FUO, 66% of the FUO cases performed FDG-PET/CT were regarded as leading to a change in the treatment plan and 25% of them were regarded as leading to improvement of the certainty factor with respect to the final cases, even though FDG-PET resulted in moderate sensitivity and specificity for the diagnosis of FUO [37].

Regarding FUO in children, a review based on 2 studies with 76 cases found that pediatric patients with abnormal PET findings were approximately 17 times more likely to achieve a definite diagnosis than those with normal PET findings (OR: 16.59, 95% CI 6.7–41) [57]. Based on a study that included 110 children with FUO, Pijl et al. reported that FDG-PET/CT showed sensitivity of 85.5% (95% CI 0.74–0.93), specificity of 79.2% (95% CI 0.65–0.90), PPV of 84.1% (95% CI 0.75–0.90), and NPV of 80.9% (95% CI 0.69–0.89) for the diagnosis of FUO. Treatment modification was made after FDG-PET/CT in 53% of the cohort, but the ratio of mortality within 3 months after FDG-PET/CT was 7%. FDG-PET/CT revealed that the cause of FUO in children was most frequently infection (57.3%) such as endocarditis, followed by NIID (37.2%) such as systemic juvenile idiopathic arthritis, miscellaneous diseases (17.9%) such as inflammatory bowel disorder as a representative disease, and neoplasm (8.4%) [58]. Accordingly, FDG-PET/CT can contribute to diagnosis in pediatric as well as adult patients with FUO.

## Results of 26 clinical FDG-PET/CT studies for the diagnosis of FUO

To evaluate the backgrounds and results in clinical studies of FDG-PET/CT for diagnosis of FUO, 26 clinical studies (Table 1) [59–84] were selected that satisfied the following conditions: (1) targeting FUO (not mixed with inflammation of unknown origin [IUO]), (2) used FDG-PET/CT for the diagnosis of FUO, (3) provided the FDG-PET/CT results for cases that were ultimately undiagnosed, and (4) sample size > 10). The performance of FDG-PET/CT in selected 26 articles were listed in Table 2. The total number of enrolled case was 1637, and the mean number of enrolled cases per study was 63 ± 57 (range 12–303) in the selected clinical studies. The majority of the clinical studies were retrospective cohorts from a single institution. Major mandatory tests in the process of diagnosing FUO were blood test, blood culture, urine test, urine culture, and blood test regarding NIID. The mandatory imaging examinations were chest X-ray, AUS, and chest and abdominal CT. Therefore, FUO patients who underwent FDG-PET/CT had remained undiagnosed throughout these diagnostic processes. The etiology of disease classification causing FUO in these studies was highest in infection (33%), following NIID (23%), malignancy (14%), and miscellaneous (3%). The ratio of FUO patients was remained undiagnosed after FDG-PET/CT was 27%. The median values of sensitivity, specificity, PPV, accuracy, and diagnostic yield were 84.5, 60, 83, 73, and 50%, respectively; calculated by the number of cases, these values were 83, 64, 81, 76, and 54%, respectively. Even in a small number of studies with a prospective cohort, specificity, PPV, and diagnostic yield were lower than in those with a retrospective cohort. The diagnostic yield was higher in studies without mandatory tests than with them, and was also higher in studies without mandatory imaging examinations than with them (Table 3). Therefore, performing laboratory and imaging tests prior to FDG-PET/CT might provide a diagnosis in some patients, and only those in whom the diagnosis is difficult would require FDG-PET/CT. Nevertheless, FDG-PET/CT could identify the source of fever in half of the remaining undiagnosed patients.

**Table 1:** Background of 26 clinical studies regarding FDG-PET/CT for the diagnosis of FUO

No.	Author	Published year	Study type	Single or multicenter	Laboratory test before FDG-PET/CT	Imaging test before FDG-PET/CT
1	Keidar et al. [59]	2008	Pros	Single	BT, UT, BC, UC	Chest X-ray, AUS or CT
2	Balink et al. [60]	2009	Retro	Single	None	None
3	Federici et al. [61]	2010	Retro	Single	BT, UT, BC, UC, BT for NIID, tuberculosis test	Chest X-ray, AUS
4	Ferda et al. [62]	2010	Retro	Single	None	None
5	Kei et al. [63]	2010	Retro	Single	None	None
6	Sheng et al. [64]	2011	Retro	Single	BT, UT, BC, UC, BT for NIID	CT, MRI, NM
7	Pelosi et al. [65]	2011	Retro	Single	BT, UT	Chest X-ray or CT, AUS or CT
8	Ergül et al. [66]	2011	Retro	Single	BT, BC, UT	Chest X-ray (CT, MRI and NM in need)
9	Kim et al. [67]	2012	Retro	Single	BT, UT, BC, UC, BT for NIID, others	Chest X-ray, AUS (CT, MRI and ECG in need)
10	Crouzet et al. [68]	2012	Retro	Single	BT, UT, BC, UC, BT for tuberculosis, others	Chest X-ray, AUS
11	Pedersen et al. [69]	2012	Retro	Single	BT, UT	None
12	Nakayo et al. [70]	2012	Retro	Single	BT, UT, BC, UC, BT for NIID, others	Chest X-ray, AUS, ECG, CT, NM
13	Manohar et al. [71]	2013	Retro	Single	BT, UT, BC, UC, BT for NIID, others	Chest X-ray
14	Tokmak et al. [72]	2014	Retro	Single	BT, UT, BC, UC, BT for NIID, others	Chest X-ray, AUS (CT, MRI and ECG, in need)
15	Gafter-Gvili et al. [73]	2014	Retro	Single	BT, BC	Chest X-ray (CT, MRI and ECG in need)
16	Bucher-Olsen et al. [74]	2015	Retro	Single	BT, BC	Chest X-ray, CT, US
17	Singh et al. [75]	2015	Pros	Single	BT, UT, BC, UC, BT for NIID	Chest X-ray, AUS, CECT
18	Pereira et al. [76]	2016	Retro	Single	BT	None
19	Hung et al. [77]	2017	Pros	Single	BT, UT, BC, UC	Chest X-ray or CT, AUS
20	García-Vicente et al. [78]	2018	Retro	Single	BT	None
21	Okuyucu et al. [79]	2018	Retro	Single	BT, UT, BC, UC, BT for NIID, tuberculosis	Chest X-ray, AUS
22	Georga et al. [80]	2020	Retro	Single	BT, BC, UC	Chest X-ray (CT, MRI, and ECG for majority of Pt)
23	Das et al. [81]	2021	Retro	Single	BT, UT, BC, UC, BT for NIID, others	Chest X-ray, AUS, ECG
24	Letertre et al. [82]	2021	Retro	Single	BT	(CT in need)
25	Buchritis et al. [83]	2021	Retro	Single	BT, BC	Chest X-ray
26	Mahajna et al. [84]	2021	Retro	Single	BT, UT, BC, UC, BT for NIID, others	Chest X-ray, AUS or CT

*Pros* prospective study, *Retro* retrospective study, *BT* blood test, *UT* urine test, *BC* blood culture, *UC* urine culture, *NIID* noninfectious inflammatory disease, *AUS* abdominal ultrasonography, *CT* computed tomography, *MRI* magnetic resonance imaging, *NM* nuclear medicine, *ECG* echocardiography, *Pt* patient

**Table 2:** Performance of FDG-PET/CT for the diagnosis of FUO by study design, mandatory test, and imaging test in 26 clinical studies

No.	Author	Enrolled cases	Disease type causing FUO (%)					Reported or calculable results						Redefining
			Infection	NIID	Malignancy	Miscellaneous	Undiagnosed	Sensitivity	Specificity	PPV	Accuracy	DY	TP/FN/FP/TN	TP/FN/FP/TN
1	Keidar et al. [59]	48	19	33	6	2	40	100	81	81	90	46	22/0/5/21	22/7/4/15
2	Balink et al. [60]	68	37	21	3	4	35	100	90	93	96	56	38/6/3/21	38/6/3/21
3	Federici et al. [61]	14	29	36	7	0	29	70	75	88	71	50	7/3/1/3	7/3/1/3
4	Ferda et al. [62]	48	38	38	17	0	8	98	75	98	96	90	43/1/1/3	43/1/1/3
5	Kei et al. [63]	12	33	8	17	0	42	71	60	71	67	42	5/2/2/3	5/2/2/3
6	Sheng et al. [64]	48	31	19	25	0	25	89	33	80	75	67	32/4/8/4	32/4/8/4
7	Pelosi et al. [65]	24	25	29	13	4	29	65	71	85	67	46	11/11/2/0	11/6/1/6
8	Ergül et al. [66]	24	13	13	21	8	46	92	45	63	79	50	12/0/7/5	12/1/6/5
9	Kim et al. [67]	48	29	4	15	38	15	89	20	61	60	52	25/3/16/4	25/16/3/4
10	Crouzet et al. [68]	79	29	25	15	8	23	98	87	96	95	74	45/19/2/13	Unclassifiable
11	Pedersen et al. [69]	22	5	41	14	0	41	67	71	83	68	45	10/5/2/5	10/4/2/6
12	Nakayo et al. [70]	20	15	15	25	0	45	79	83	92	80	55	11/3/1/5	12/8/0/0
13	Manohar et al. [71]	103	30	21	13	3	33	90	97	98	92	60	62/7/1/3	62/7/1/3
14	Tokmak et al. [72]	25	32	40	12	0	16	94	80	94	90	71	15/1/1/4	15/6/1/3
15	Gafter-Gvili et al. [73]	112	44	15	13	2	26	72	58	75	65	46	52/20/17/23	51/32/9/20
16	Bucher-Olsen et al. [74]	57	51	21	7	7	14	93	60	68	75	44	25/2/12/18	25/24/3/5
17	Singh et al. [75]	47	19	21	11	2	47	82	32	51	55	38	18/4/17/8	18/7/14/8
18	Pereira et al. [76]	76	21	12	22	5	40	77	31	61	58	45	32/14/0/5	43/3/10/20
19	Hung et al. [77]	58	40	19	17	3	21	79	56	83	72	57	33/9/7/9	33/13/4/8
20	García-Vicente et al. [78]	67	31	49	6	1	12	84	31	62	61	48	32/6/20/9	32/26/0/9
21	Okuyucu et al. [79]	74	26	45	9	5	14	75	69	92	74	62	46/15/4/9	46/19/0/9
22	Georga et al. [80]	50	40	22	16	0	22	95	50	84	84	72	36/2/6/6	36/4/4/6
23	Das et al. [81]	43	23	37	9	5	26	77	33	83	51	47	20/6/4/2	20/12/11/0
24	Letertre et al. [82]	39	23	44	8	5	21	85	37	58	62	44	17/3/12/7	17/3/12/7
25	Buchritis et al. [83]	303	37	17	18	0	28	89	82	87	86	52	158/20/23/102	158/20/23/102
26	Mahajna et al. [84]	128	48	16	9	1	26	70	37	70	67	47	60/16/26/26	60/33/9/26

*NIID* noninfectious inflammatory disease, *PPV* positive predictive value, *DY* diagnostic yield, *TP* true positive, *FN* false negative, *FP* false positive, *TN* true negative

**Table 3:** Performance of FDG-PET/CT for the diagnosis of FUO based on literature and redefining in 26 clinical studies

Index		Median					Result based on literature					Redefined				
		Sensitivity	Specificity	PPV	Accuracy	DY	Sensitivity	Specificity	PPV	Accuracy	DY	Sensitivity	Specificity	PPV	Accuracy	DY
All 26 studies		84.5	60	83	73	50	83	64	81	76	54	75	71	86	74	53
Study design	Prospective	82	56	81	72	46	85	57	72	73	48	73	58	77	68	48
	Retrospective	85	60	83	74	50	82	64	82	76	55	76	73	87	75	54
Laboratory test before PET/CT	None	98	75	93	96	56	91	82	93	88	67	91	82	93	88	67
	BT	77	37	62	62	45	72	42	73	63	50	73	65	82	71	50
	BT and BC	92.5	59	82	81.5	51	84	71	83	85	56	77	75	86	83	56
	BT, BC, BT for NIID	80.5	53	85.5	72.5	53.5	72	65	86	70	54	83	55	79	74	55
Imaging test before PET/CT	None	80.5	65.5	77	67.5	46.5	82	62	85	77	60	80	78	90	80	58
	Chest X-ray and/or AUS	83	78.5	90	80	56	83	82	91	83	56	83	80	89	82	55
	CT	87	53	77.5	73.5	48.5	82	51	73	70	51	69	60	83	67	51

*PPV* positive predictive value, *DY* diagnostic yield, *BT* blood test, *BC* blood culture, *NIID* noninfectious inflammatory disease, *AUS* abdominal ultrasonography, *CT* computed tomography

In these studies, the definitions of false negative (FN), FP, and TN for FDG-PET/CT were not unified. In the case that FDG-PET/CT showed pathological findings that did not relate to the cause of fever, the case was commonly regarded either as FP (identified an area not related to the cause of FUO) or FN (could not identify the cause of FUO). Therefore, for further analysis, true positive (TP), FN, FP, and TN are redefined as follows: TP: FDG-PET/CT findings confirmed pathologically as the cause of the fever; FN: cause of fever unidentifiable by FDG-PET/CT in the case of confirmed pathological diagnosis; FP: positive FDG-PET/CT findings in the case of undetermined pathological diagnosis; and TN: etiology of fever undetermined both pathologically and on FDG-PET/CT.

The sensitivity, specificity, PPV, accuracy, and diagnostic yield of FDG-PET/CT were 75, 71, 86, 74, and 53%, respectively, calculated by the number of cases (Tables 2, 3).

## FDG-PET/CT protocol and possible factors related to diagnostic results

A basic FDG-PET/CT examination protocol for investigation of FUO is provided in the European Association of Nuclear Medicine/Society of Nuclear Medicine and Molecular Imaging (EANM/SNMMI) guidelines [85]. Considering the attributes of FDG-PET/CT imaging for detection of background diseases causing FUO, whole-body imaging is performed from vertex to toe. A deficiency of clinical information in patients with FUO may suppress the diagnostic performance of FDG-PET/CT [41]. The administration of antibiotics appears to have no clinically significant impact on the diagnostic accuracy of FDG-PET/CT performed for evaluation of known or suspected infectious processes, whereas administration of corticosteroids may lead to false-negative results in the case of FUO caused by systemic disease. [86]. Tsuzuki et al. reported that the diagnostic yield of FDG-PET/CT in patients with FUO or IUO was not related to age, sex, symptom duration, maximum body temperature, CRP, WBC count, ESR, Hb, or sIL-2 [87]. Garcia-Vincente et al. found that FDG-PET/CT results (positive or negative) were associated with the final diagnosis and with positive culture results, and that positive inflammation markers and protein analysis alterations were potent factors but not significantly so. Regarding the diagnostic performance of FDG-PET/CT, three or more positive inflammation markers and pathologic protein analysis were potent factors but not statistically significant, and maximum body temperature and positive serology were not related to the performance of FDG-PET/CT [78]. Crouzet et al. found that CRP > 30 mg/L and anemia were significantly associated with a performance of PET. The success rate of FDG-PET/CT was significantly higher in patients with constitutional symptoms and with suspected malignancy, whereas age, sex, duration of fever, CRP, leukocytes, and previous antibiotic treatment were not related to the success rate of FDG-PET/CT [68].

Balink et al. reported that elevated CRP levels < 20 mg/L were more predictive of positive FDG-PET/CT than ESR levels < 20 mm/h, and that FDG-PET/CT was 100% true negative in patients with CRP levels < 5 mg/L [88]. In another report, the diagnostic value of FDG-PET/CT increased in the conditions of presence of fever on the day of the scan and of the presence of elevated CRP within 7 days before the scan [89].

Regarding children with FUO, CRP was positively but weakly associated with identification of a true positive focus of fever on FDG-PET/CT (OR = 1.01 (95% CI 1.00–1.02) per mg/L increase in CRP) [58]. Overall, no significant index appears to be related to the diagnostic yield of FDG-PET/CT.

**Fig. 3:**
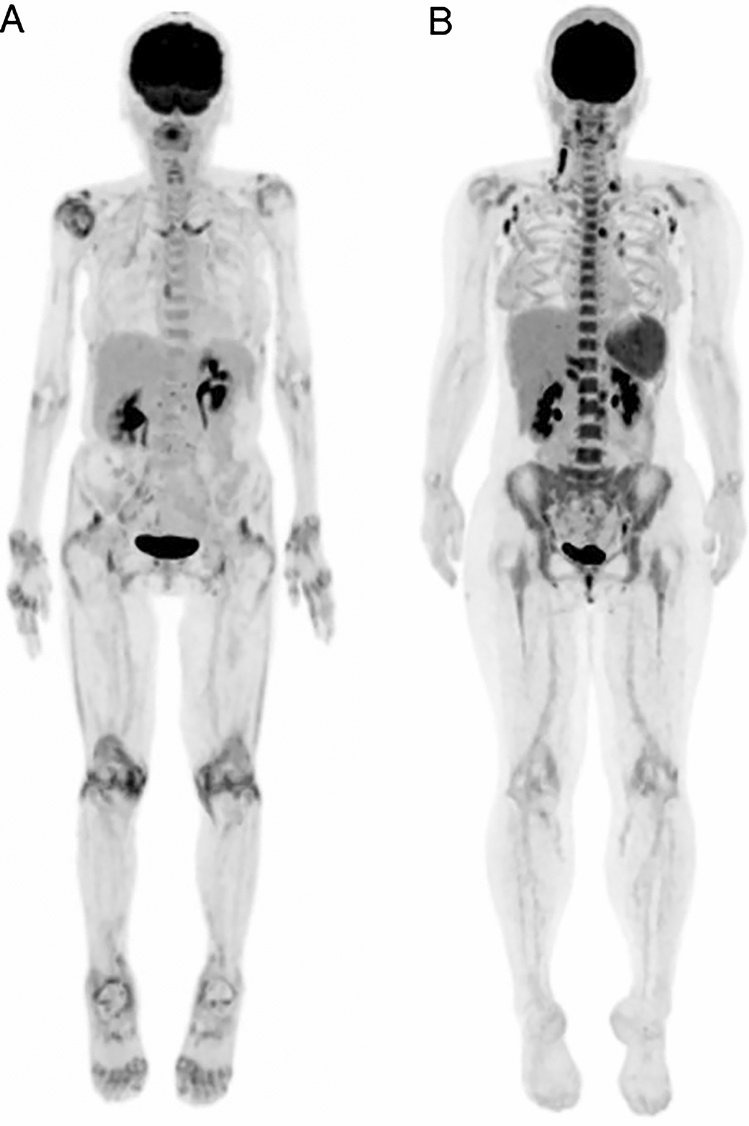
Typical FDG-PET imaging feature of PMR and AOSD. **A** Polymyalgia rheumatica (PMR): FDG uptake is confirmed at ischial tuberosity, greater trochanters, interspinous bursae, hips, shoulders, and sternoclavicular joints. **B** Adult-onset Still’s disease (AOSD): intense FDG uptake is confirmed at bone marrow, lymph nodes, and spleen

Because qualitative assessment is performed for the identification of any pathological FDG uptake related to the cause of prolonged fever, sites of para-physiological radiopharmaceutical uptake may be missed on FDG-PET/CT [56]. False-negative FDG-PET or PET/CT results can occur in systemic lupus erythematosus, cytomegalovirus infection, toxoplasmosis, urinary infection, septicemia, pyelonephritis, and Crohn’s disease [90]. PMR and AOSD have been reported as the major causes for which FDG-PET/CT most often showed no pathologic uptake leading to diagnosis [39](Fig. 3).

According to the 2012 provisional classification criteria for polymyalgia rheumatica, several disease symptoms and laboratory test results (CRP, ESR, rheumatoid factor, and anti-cyclic citrullinated peptide antibody) and findings have been proposed in the US as the criteria for classification of PMR [88], but are not intended for diagnostic use [91]. Although fever can occur in patients with PMR, it is not included in the criteria as a symptom indicating PMR; rather, GCA should be suspected [90]. A pathological diagnosis is not a decisive factor in the diagnosis of PMR. FDG-PET/CT is useful for the diagnosis of PMR because of the specific findings of FDG uptake at the ischial tuberosity, greater trochanters, interspinous bursae, hips, shoulders, and sternoclavicular joints [92, 93]. Therefore, FDG uptake distribution patterns and morphology can support a definitive diagnosis of PMR, and it is also useful for differentiating between PMR and elderly onset rheumatoid arthritis. However, the site of FDG accumulation may not always correlate with the clinical symptoms, and there is a possibility of subclinical conditions and the spread of other inflammation.

“Fever of at least 39 °C for at least a week” is one of the major diagnostic criteria of AOSD [94]. The diagnosis of AOSD is often difficult and time-consuming because of the absence of specific clinical, radiological, biological, and pathological characteristics [95]. The typical FDG-PET/CT findings in AOSD are uptake in bone marrow, lymph nodes, and spleen, and the intensity of FDG uptake is correlated with disease activity [96]. The differential diagnosis for AOSD includes infectious disease, malignant disease, and systemic disease [97], and lymphoma and viral infections that can cause FUO show a similar pattern of FDG uptake as for AOSD. The pathological findings of AOSD in lymph nodes are lymphadenitis or lymphatic hyperplasia to varying degrees. Even though FDG-PET/CT can identify pathological lymph nodes and lead to biopsy, the low specificity of the pathological findings is less useful for the diagnosis of AOSD [98], even though it is significant to exclude the diagnoses of malignant lymphoma and infectious lymphadenitis, which should be differentiated in the diagnostic process of AOSD. Therefore, FDG-PET/CT has limitations in the definitive diagnosis of AOSD in terms of image interpretation.

## Outcomes in patients with FUO after FDG-PET/CT

In patients with FUO who obtain a final diagnosis by FDG-PET/CT, the prognosis depends on the underlying disease. FDG-PET/CT has been reported to reduce mortality rates in patients with infection such as staphylococcus aureus bacteremia, Gram-positive bacteremia, and pacemaker or defibrillator infection [99–101]. Therefore, FDG-PET/CT may have been a gamechanger in leading to a better prognosis for patients with FUO, because it enables an earlier final diagnosis and earlier start of appropriate treatment than before. A certain percentage of FUO patients remains undiagnosed after FDG-PET/CT. Patients with negative FDG-PET/CT results were significantly more likely to present with spontaneous regression than those with positive results (summary RR = 5.6; 95% CI 3.4–9.2) [55], Therefore, observation can also be considered an option in the clinical process for undiagnosed FUO patients in the event that FDG-PET/CT shows no specific findings.

## Cost-effectiveness

Nakayo et al. analyzed cost-effectiveness in the diagnosis of FUO and estimated the influence of FDG-PET/CT on this cost. The real cost of the FUO process before the PET/CT test was 11,167.35€ per patient, which included 9620.82€ for 28.15 days of hospitalization, 151.63€ for 0.85 days of outpatient consultation, and 1394.90€ for complementary tests through diagnostic phases I–III and other tests. In contrast, the total theoretical cost of the FUO process before the FDG-PET/CT test was 7433.50€ per patient, which includes 5639.20€ for 16.50 theoretical hospitalization days, 178.78€ for 1 day of outpatient consultation, and 1615.52€ for complementary tests through diagnostic phases I–III. When FDG-PET/CT is performed in phase II (end of the second diagnostic week) in the FUO process, the theoretical cost of the entire FUO process is estimated to be 5696.22€, consisting of 4443.01€ for 13 theoretical days of hospitalization, 178.78€ for 1 day of outpatient consultation, and 1074.43€ for complementary tests through two diagnostic phases including FDG-PET/CT (500.00€). As a result, 5471€ per patient was estimated to be saved per patient [70].

Chen et al. evaluated the cost-effectiveness of FDG-PET/CT for FUO and IUO patients. The mean medical costs of the FDG-PET/CT group (23,556.96¥ [≒3337.47€]) were significantly higher than those of the non-FDG-PET/CT group (9266.65¥ [≒1312.87€]). However, a higher rate of definite diagnosis was achieved in the FDG-PET/CT group (91.4%) than in the non-FDG-PET/CT group (86.5%). The mean hospitalization days and mean medical costs before diagnosis were significantly lower in patients who had undergone FDG-PET/CT within 7 days after hospital admission than in those at 8 days or more after admission [102]. In another study of patients with IUO, the diagnostic rate was higher (with FDG-PET/CT: 70%, without FDG-PET/CT 30%) and cost per patient was lower (with FDG-PET/CT: 5298€, without FDG-PET/CT: 126,143€) in those who received FDG-PET/CT than in those who did not [103]. Accordingly, FDG-PET/CT appears to be cost-effective for patients with FUO, and efficiency might be improved if it is performed in a relatively early phase of the diagnostic process for FUO.

## Status of clinical application of FDG-PET/CT for patients with FUO

The “Guideline for ^18^F-FDG Use in Inflammation and Infection” published for use in Europe and the United States state the usefulness of diagnosing inflammatory diseases [84]. The core SmPC and package leaflet guidelines for fludeoxyglucose (^18^F) published by the European Medicines Agency (EMA) documents “Localisation of abnormal foci guiding the aetiologic diagnosis in case of fever of unknown origin” as an indication for FDG. In the United Kingdom, Germany, and France, FDG has been designated as a “localization diagnosis of abnormal lesions that guides pathological diagnosis in fever of unknown origin” [104]. In the US, following the huge effort of SNMMI in approaching the Centers for Medicare and Medicaid Services (CMS), the CMS retired the National Coverage Determination (NCD) for ^18^F-FDG-PET for infection and inflammation (including FUO), which was effective on 1 January 2021, and updated in August 2021. As a result, coverage determinations for PET for infection and inflammation will be made at the discretion of local Medicare Administrative Contractors (MACs) [105, 106]. In Japan, FDG-PET/CT is not covered by national health insurance, but a prospective survey in Japan reported that FDG-PET/CT examinations were used in self-financed medical care for about 31% of patients with fever of unknown origin [22], and it is considered that this application will continue to progress. In the past 10 years, the availability of FDG-PET/CT has greatly expanded due to supporting clinical evidence. The appropriate use of FDG-PET/CT for patients with FUO is therefore expected to increase in the future.

## FDG-PET/CT applied to the diagnosis of IUO

The term IUO is defined as FUO with a temperature not exceeding 38.3 °C, accompanied by elevated inflammatory markers on several occasions. The diagnostic approaches used for IUO are identical to those recommended for FUO [107]. The review of Affronni et al. found that the diagnostic approach taken for patients with FUO can also be applied to patients with low-grade fever (body temperature of 37.5–38.3 °C) on the basis that there was no relationship between body temperature values and severity of the underlying disease, and the disease spectrum was identical to that of FUO [108]. Vanderschueren et al. reported that the diagnostic yield, case mix, contribution of FDG-PET, and vital outcomes were similar between IUO and FUO [109]. FDG-PET/CT has shown comparable diagnostic sensitivity, specificity, and accuracy for IUO as for FUO [110–112]. In addition, numerous clinical studies that analyzed the performance of FDG-PET/CT for the diagnosis of combined FUO and IUO patients have reported similar utility of FDG-PET/CT as that of FUO patients alone [113–122].

## Conclusion

FDG-PET/CT can be applied to patients with an unknown cause of prolonged fever despite basic diagnostic workup. High sensitivity and relative specificity have been reported, and diagnostic yield is expected for over half of FUO patients. In patients who remained undiagnosed after FDG-PET/CT, those with negative FDG-PET/CT results tend to have frequent spontaneous regression of fever. The availability of FDG-PET/CT has greatly expanded in the past 10 years, supported by clinical evidence. Based on this knowledge, the appropriate use of FDG-PET/CT for future patients with FUO is expected.
